# Structural Immunology of Complement Receptors 3 and 4

**DOI:** 10.3389/fimmu.2018.02716

**Published:** 2018-11-26

**Authors:** Thomas Vorup-Jensen, Rasmus Kjeldsen Jensen

**Affiliations:** ^1^Biophysical Immunology Laboratory, Department of Biomedicine, Aarhus University, Aarhus, Denmark; ^2^Interdisciplinary Nanoscience Center, Aarhus University, Aarhus, Denmark; ^3^Department of Molecular Biology and Genetics—Structural Biology, Aarhus University, Aarhus, Denmark

**Keywords:** innate immunity, complement, complement receptors, integrins, cell adhesion, von willebrand facor A (VWA) domain, divalent metal ions, drug repurposing

## Abstract

Complement receptors (CR) 3 and 4 belong to the family of beta-2 (CD18) integrins. CR3 and CR4 are often co-expressed in the myeloid subsets of leukocytes, but they are also found in NK cells and activated T and B lymphocytes. The heterodimeric ectodomain undergoes considerable conformational change in order to switch the receptor from a structurally bent, ligand-binding in-active state into an extended, ligand-binding active state. CR3 binds the C3d fragment of C3 in a way permitting CR2 also to bind concomitantly. This enables a hand-over of complement-opsonized antigens from the cell surface of CR3-expressing macrophages to the CR2-expressing B lymphocytes, in consequence acting as an antigen presentation mechanism. As a more enigmatic part of their functions, both CR3 and CR4 bind several structurally unrelated proteins, engineered peptides, and glycosaminoglycans. No consensus motif in the proteinaceous ligands has been established. Yet, the experimental evidence clearly suggest that the ligands are primarily, if not entirely, recognized by a single site within the receptors, namely the metal-ion dependent adhesion site (MIDAS). Comparison of some recent identified ligands points to CR3 as inclined to bind positively charged species, while CR4, by contrast, binds strongly negative-charged species, in both cases with the critical involvement of deprotonated, acidic groups as ligands for the Mg^2+^ ion in the MIDAS. These properties place CR3 and CR4 firmly within the realm of modern molecular medicine in several ways. The expression of CR3 and CR4 in NK cells was recently demonstrated to enable complement-dependent cell cytotoxicity toward antibody-coated cancer cells as part of biological therapy, constituting a significant part of the efficacy of such treatment. With the flexible principles of ligand recognition, it is also possible to propose a response of CR3 and CR4 to existing medicines thereby opening a possibility of drug repurposing to influence the function of these receptors. Here, from advances in the structural and cellular immunology of CR3 and CR4, we review insights on their biochemistry and functions in the immune system.

## Introduction

Complement receptors (CRs) make an important link between cellular functions, notably—but not exclusively—between functions of the leukocytes and soluble complement components (C), factors (F), and several related proteins. They also create a strong connection between those parts of the immune system often termed the innate immune system and other parts forming the adaptive immune system. Among these receptors, especially CR3 has been the subject of several studies at essentially all levels of modern biology including its biochemistry to *in vivo* analysis by use of transgenic mice (1). Nevertheless, in spite of more than 40 years of research, the full versatility of CR3 seems not to have been captured as yet, not to mention the structurally similar CR4, which is even less understood.

The present review focuses on highlighting both a few past and some more recent insights on the structural biology and functions of CR3 and CR4. The focus is on extracellular biology of these receptors, comparing their ligand recognition and how to put their structural biology into a context of immunology. The equally important, and quickly developing, topic, of intracellular signaling by CR3 and CR4 is only briefly touched upon. The Reader is referred to other authoritative reviews for a more comprehensive elucidation of this topic (2–4).

It is not a new idea to review the literature on CR3 and CR4 together (5–7). The present paper aims to make a critical contribution by addressing the question why we have come to think of these receptors as particularly similar. To this end, the present section includes a brief historical summary on the discovery of CR3 and CR4, followed by a broader introduction to their family of β_2_ (CD18) integrins. Section the structure, conformational regulation, and ligand recognition by CR3, and CR4 addresses the conundrum of CR3 and CR4 ligand recognition in the context of advances in the structural biology of these receptors. In Section Therapeutic interventions targeting CR3 and CR4, an obvious, yet in the literature surprisingly absent, theme is brought up, namely what role CR3 and CR4 play in human medical therapy. The current situation is paradoxical as no medicines in use are directed to these receptors, but several pharmacological agents may nevertheless target CR3 and CR4 functions, at least as conjectured from primarily biochemical and cellular investigations. Finally, Section Conclusion: CR3 and CR4, significant contributors to both innate and adaptive immunity concludes by looking ahead to the next important steps in the investigations of CR3 and CR4.

### Functions of CR3 and CR4 and the family of CD18 integrins

CR3, at the time named Mac-1, was discovered by Springer et al. (8). They immunized rats with a human leukocyte cell membrane extract and thereby produced a monoclonal antibody (Ab), the M1/70, which was the first to react with a “discrete molecule specific to phagocytes” (8). The activity toward phagocytes prompted the question of the M1/70 impact on complement-opsonized phagocytosis. Indeed, M1/70 blocked the interaction of neutrophils with iC3b (9), an activity assigned before as constituted by CR3 but with no molecule “in hand” (10).

The discovery of CR4 was more convoluted. Originally characterized as a part (p150,95) of the product in pull-down experiments with Ab to CD18, little information was obtained on its function initially (11). By the use of affinity matrices coupled with iC3b, it was possible to pull down the p150,95 antigen (12, 13). The similarity in terms of ligand specificity with CR3 was striking (13), also including the inability of CR4 to react with C3d, an observation, which has received further support recently (14). CR4 has been useful as a widely employed marker of murine dendritic cells (with the nomenclature CD11c/CD18) following the observation that this molecule is the most abundant in the cell membrane of these cells (15). A remarkable property of both CR3 and CR4 is the intracellular location of receptors stored in neutrophil granula (11). Upon activation of neutrophils, for instance using the bacterial product *N*-formylmethionine-leucyl-phenylalanine, CR3 is particularly mobilized from these storages to the cell membrane functionally enabling these cells to respond to iC3b deposited on targets (16–18). This provides an ~17-fold upregulation of expression in the membrane through a mechanism which has no immediate transcriptional component. CR3 provides an example sometimes overlooked in the age of transcriptomics, that not all protein expression is regulated by mRNA synthesis and decay, at least in the cell membrane.

From a protein phylogenetic standpoint, CR3, and CR4 belong to the β_2_ integrin family of adhesion molecules (19, 20). The family contains four members, namely integrins α_L_β_2_ (lymphocyte function-associated antigen [LFA]-1 or CD11a/CD18), α_M_β_2_ (Mac-1, CR3, or CD11b/CD18), α_X_β_2_ (CR4, p150,95 or CD11c/CD18), and α_D_β_2_ (CD11d/CD18). CD18 integrin expression is restricted to leukocytes and one or more types are found on nearly all leukocytes (19). An interesting exception is human and murine mast cells, which as part of the maturation process lose CD18 expression (21, 22). The functional consequences of this situation remain unknown.

LFA-1 is expressed in both lymphocytes and myeloid cells, while CR3 and CR4 is strongly expressed in macrophages and non-classical monocytes, which are usually considered a precursor cell of the tissue-embedded macrophages. Neutrophil granulocytes are also prominently expressing CR3 and CR4 (23). The expression in lymphocytes is more varying and probably dependent on activation. Natural killer (NK) cells express high levels of LFA-1, CR3 and CR4 (19). A less strong expression of CR3 and CR4 is also found in some T and B cells as reported in a few studies (24–26). The integrin α_D_β_2_ is not particularly well studied in any respect, but the expression and functions seems to share some properties with CR3 and CR4 (27, 28).

CD18 integrins serve important roles in leukocyte cell contacts. They are involved in the extravasation of leukocytes through the endothelium to zones of inflammation, the contact between lymphocytes and antigen presenting cells, and phagocytosis of complement opsonized targets.

LFA-1 is the key molecule in regulating the contacts of leukocytes with intercellular adhesion molecule (ICAM)-1 expressed on activated endothelium (29–31). CR3 may also interact with ICAM-1(32) and LFA-1, CR3, and CR4 have been reported to bind ICAM-4 (33, 34). However, it remains unclear whether these interactions are auxiliary to LFA-1-mediated adhesion or serve purposes that are more specialized. The LFA-1/ICAM-1 interaction also serves the important task of the formation of the immunological synapse (19, 35), crucial the contact between antigen presenting cells (APC) and T lymphocytes. On the surface of the APC, ICAM-1 molecules will form bonds to LFA-1 on the T lymphocyte, essentially in an outer circle surrounding the T cell receptor (TCR)-major histocompability complex (MHC) molecule. This organization easily follows from the curvature of the cells and the longer stretch of the LFA-1/ICAM-1 compared to the TCR-MHC complex (36). Interestingly, it is not known if CR3 and CR4 forms similar structures as part of their contact with target surfaces. If their ligands on such a surface is complement, the deposition could present less order in the spatial organization of CR3 and CR4 ligands than what is seen for the classic immunological synapse. On the other hand, results from nanomicrobiology have pointed to a high level of surface structure of the microbial cell wall. For instance, the peptidoglycan cell wall of *Staphylococcus aureus* was shown to be built in concentric circles (37). Likewise, the fungal human pathogen *Aspergillus fumingatus* also present woven textile-like surface pattern (38). The patterns probably affects the binding of certain polyvalent immune effector molecules such as IgM and mannan-binding lectin (MBL) (39, 40). However, it seems plausible that deposition of molecules such as the complement component C3-fragment iC3b could be guided by the surface structure. With the concentric ridges on the surface of *S. aureus*, it is not inconceivable that this would impose ring-like organization of CR3 or CR4 in the leukocyte cell membrane upon contact with the complement-opsonized bacterial surface. Investigations on these questions are lacking.

As mentioned above, NK cells are carrying high levels of CR3 and CR4. A few studies in the past documented their role in complement-dependent cell cytotoxicity (CDCC), but the role of this NK cell effector mechanism was unclear, at least compared to the better understood antibody-dependent cell cytotoxicity (ADCC). A recent study has now shown that CDCC may account for as much as 50% of NK cell cytotoxicity to anti-CD20 (rituximab)-covered B cell targets (41). Of course, this finding opens up for a better understanding of complement in antibody-based biological therapy and certainly highlights the role of CR3 and CR4 in this process. Likewise, it is also possible that the contribution of complement to certain pathologies can be now be thought of as involving NK cell cytotoxicity, including diseases with autoreactive antibodies. Especially in the latter case, the means of actually targeting the function of CR3 and CR4 appears equally important as discussed further in Section Therapeutic interventions targeting CR3 and CR4.

### Soluble CD18 complexes

An increasing number of reports have now identified shed ectodomains of CD18 integrins in the blood of humans and in mice. Mechanistically the shedding probably involves matrix metalloproteinases (MMPs), notably MMP-9 (42), although experiments in mice suggest a more complex situation, probably with several sources of pericellular proteolysis involved (43).

In humans, an initial study identified soluble (s) CD18, mainly in the form of sLFA-1, in fluid from induced blisters with large influx of neutrophil granulocytes. On these cells, a stub remained of the CD11a chain, which apparently was more degraded than the CD18 chain (44). Gjelstrup *et al*. published the analysis of three groups of arthritis patients, namely, rheumatoid, spondylo, and osteoarthritis. The sCD18 in synovial fluid from the inflamed rheumatoid and spondyloarthrtitis patients was clearly higher than the plasma concentration (44, 45). Interestingly, the plasma sCD18 concentration has turned out often to be lower in chronically inflamed patients (45, 46). This probably connects to the observation that the sCD18 species are ligand binding active to a level where they may compete with cellular adhesion as shown in several experiments with ICAM-1 as a ligand for sLFA-1 (45, 46). The most abundant type of sCD18 species in humans seem to contain the CD11a chain (45), meaning that ICAM-1 is likely the major ligand for sCD18 (47). One important observation made, so far only by Gjelstrup et al. is the oligomeric state of the sCD18 species (45). It is likely that the oligomerization enables a strong, polyvalent interaction with ligand-coated surfaces such as the tested surfaces with ICAM-1 (44–46) and iC3b (48). It was observed that recombinant sCR3 fragments oligomerize (49), but the relationship between these oligomers (45) with the oligomer forms found in plasma remains unclear. Furthermore, the structure of sCD18 oligomers, is not well-understood, not even at a level of understanding the stoichiometry of alpha and beta chains. Direct detection of sCR3 and sCR4 forms in human plasma was attempted by Gjelstrup et al. (45). Probably as the first, this study reported on barely detectable amounts of sCR3 in human plasma, later supported by reports by others demonstrating the shedding of CR3 (50). Recently, it was possible to demonstrate the antagonistic influence of full plasma on cell adhesion to iC3b, i.e., a CR3 and CR4 ligand, with reduction of the antagonism when sCD18 species were depleted (48). CR3 appears to bind the zymogen form of MMP-9 and also associates with the active enzyme in the cell membrane (51), a finding which undoubtedly has implications for receptor shedding. In murine serum, sCR3 is considerable easier to detect (52). Other studies demonstrated that shedding of CR3 is critical for the efflux of macrophages in an experimental murine model of peritonitis and presented vidence that sCR3 may act as soluble antagonist to CD18 integrin binding to ICAM-1, fibrin, and collagen (43). Attempts to measure sCR4 in human plasma failed and no reports on such species are apparently available (45). Both in this case as well as concerning the issues in making strong detection of sCR3 in human plasma, factors such as proteolytic degradation or affinity of the tested antibody recognition could explain the lack of signal.

## The structure, conformational regulation, and ligand recognition by CR3 and CR4

Both CR3 and CR4 have been helpful molecules in understanding the structural biology of integrins. The atomic-resolution structure of the CR3 ligand binding domain and the CR4 ectodomain explained critical aspects of integrin ligand binding activity and the large conformational changes enabling ligand binding. With the recent structure of a complex between the CR3 ligand binding domain and C3d, new light has been shed on how this receptor binds what is likely its most prominent ligand. In direct structural comparison between the ligand binding domains of CR3 and CR4, it is also evident why CR4 is not able to bind C3d similar to CR3, this way distinguishing the binding of C3 fragments by CR3 and CR4.

### Structure of CR3 and CR4 ectodomains

As members of the CD18 integrins, CR3 and CR4 form a heterodimeric complex containing one CD18 beta chain (β_2_) and either of the alpha chains α_M_ or α_X_, respectively. The β_2_ chain is a moderately glycosylated molecule with a *M*_r_ of 95,000. The alpha chains vary between *M*_r_s of 150–170,000, with the α_X_ being notably less glycosylated than the other chains (19). It is not known if and how the reduced glycosylation of CR4 affects its function, and the topic is not pursued further here. The CD18 integrins also contain multiple metal ion binding sites, some with significant implications for the function of the integrins (53, 54).

**Figure 1:**
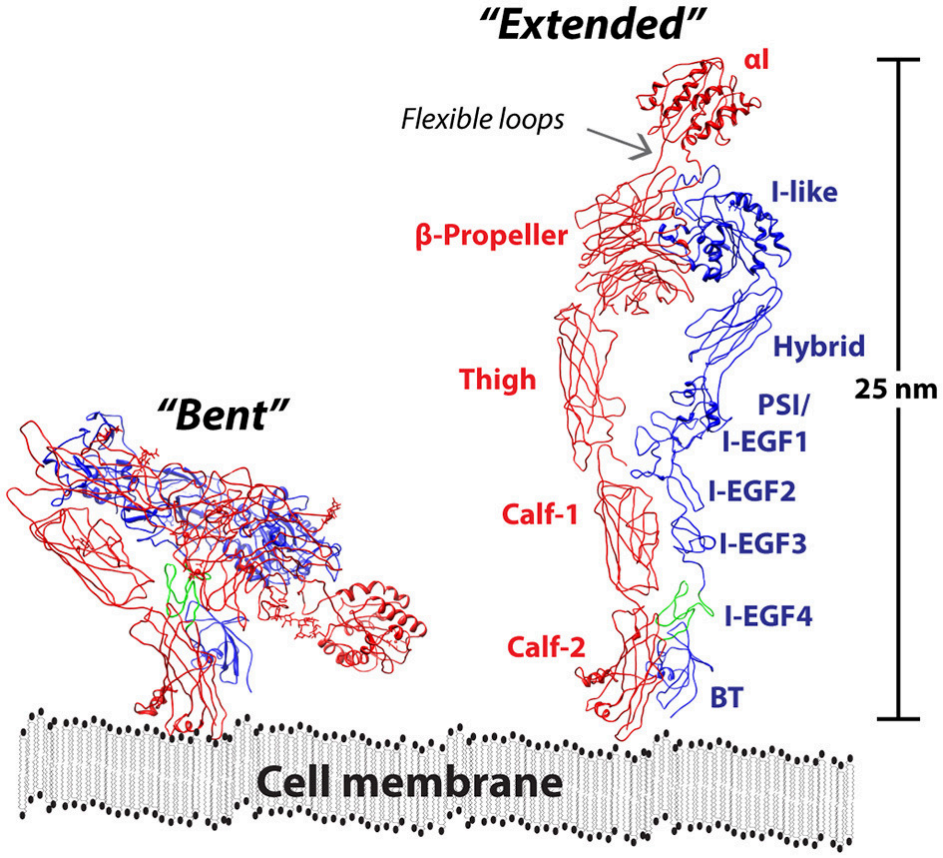
Model of the CD11/CD18 ectodomain. The CR4 in the bent conformation as determined by XRC [RSCB entry 3K72; (55)] is shown together with a model of the extended conformation. The α_X_ chain is indicated in a red color together with labels showing the approximate positions of the αI, β-propeller, Thigh, Calf-1, and Calf-2 domains. In the β_2_ chain, indicated in a blue color, the I-like, Hybrid, plexin-semaphorin-integrin (PSI), integrin epidermal growth factor (I-EGF) 1-4, and beta tail (BT) domains are shown. The ecto domain is shown in proximity to cell membrane, drawn to scale with a thickness of 3 nm (56). Reprinted from Gjelstrup et al. (57), Copyright (2011), with permission from Elsevier.

The structural organization of the CD18 integrins follows a widely conserved domain organization, also found in other integrins (Figure 1). The CD18 integrins belongs to the class of inserted (I) domain-carrying receptors. As suggested by the name, the I domain is inserted between blade W2 and W3 of the seven-bladed beta-propeller domain (58). It belongs to the family of von VWA domains, taking the Rossmann fold (59). In the CD18 integrins, the domain contains seven amphipathic helices surrounding a hydrophobic β-sheet core. This domain is found in GTPases as well as in several other molecules with adhesive functions, and notably some parasite proteins found in *Plasmodium falciparum* and *Toxoplasma gondii* are considered for use as vaccine antigens (60, 61).

**Figure 2:**
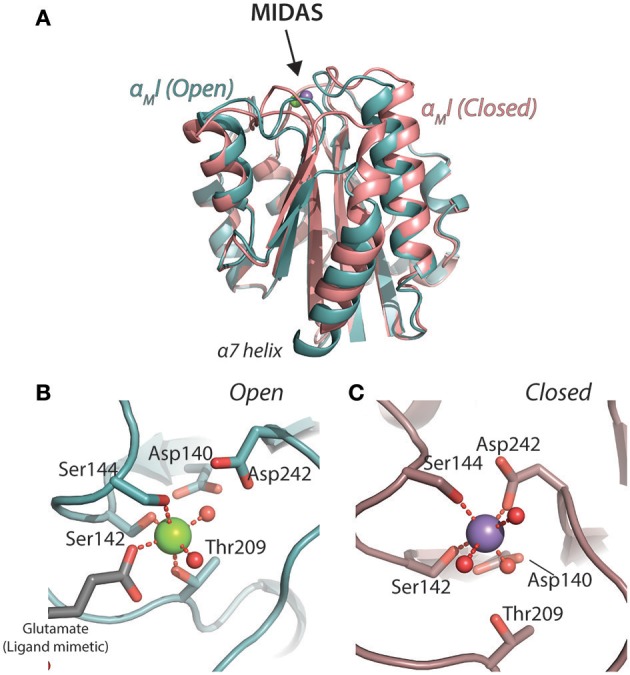
Structures of the open [1IDO; (65)] and closed [1JLM; (64) 9 α_M_I. The open structure backbone is indicated in turquoise and closed structure in a light red. **(A)** Superimposition of the structures with an indication of the MIDAS. **(B,C)** Details of the metal ion coordination spheres in the open **(B)** and closed **(C)** conformations.

The I domain is the best and most widely characterized part of the CD18 integrin structure. In those integrins carrying an I domain, it is the major ligand binding site. Isolated domains from α_L_ (62, 63), α_M_ (64–67), and α_X_ (68) have all been characterized at high resolution with X-ray crystallography (XRC). So far, a high-resolution solution structure has only been obtained for the α_L_ I domain (α_L_I) was by nuclear magnetic resonance spectroscopy (69, 70). Earlier, NMR was also used to confirm the folded nature of the α_M_I (71). Several of the key structural findings came from analysis of the α_M_I. The first structure, referred to as the “open” conformation identified the metal-ion dependent adhesion sites (MIDAS), which chelates a Mg^2+^ ion in the primary coordination sphere through the hydroxyl groups of the residues Ser142, Ser144, and Thr209 (Figure 2). Two water molecules and Glu314 from a neighboring α_M_I completed the Mg^2+^ coordination sphere (65). Another structure of the α_M_I, referred to as the “closed” conformation, showed a more compact packing of the C-terminal α7 helix and a primary coordination sphere consisting of Ser142, Ser144, Asp242, and three water molecules (64) (Figure 2). Evidently, this suggests a mechanism for regulation of ligand binding, where the side chains in the coordination sphere of the “open” conformation enables the chelation of external anionic ligands, e.g., a glutamate side chain carboxylate. Experimental evidence from both computational stabilization of the domain in the open conformation (72), a structure-guided mutation in a hydrophobic pocket in the wild-type domain keeping the C-terminal alpha helix in position (67), as well as from engineered disulphide bridges locking the domain in the open conformation confirmed that the open-conformation α_M_I had a several fold higher affinity for ligand than the closed conformation (73). It is noteworthy that the hydrophobic-pocket mutation also stabilized the α_X_I in the ligand-binding conformation (68), while the α_L_I requires a different set of mutations to be stabilized in this conformation (74).

Evidence for ligand contacts outside the I domain in CD18 integrins is limited to studies on CR3 and iC3b binding. Mutations in the alpha chain beta-propeller domain showed a reduced binding to this ligand (75). Deletion of the α_M_I from the alpha chain produces a construct that more moderately supports iC3b binding, which, however, was not completely ablated by deletion of the I domain, again supporting a ligand binding site outside the I domain (76). The involvement of the CR3 alpha chain beta-propeller domain in binding iC3b recently received further support from analysis by electron microscopy (EM) (49). A more complex aspect is the apparent ability of CR3 to interact with certain carbohydrate chains, notably β-glucan (77, 78). A well-defined binding site for this interaction has not been characterized, even though some evidence from a function-blocking antibody suggest a location in the membrane proximal part of the α_M_ chain (77). The interaction seems to be able to prime certain anti-cancer responses (77), but the mechanistic part remains uncertain, including the possible involvement of a lectin co-receptor in complex with CR3.

With regard to the function of the CD18 integrin ectodomain outside the I and beta propeller domains, a wide range of experimental work on several types of integrin receptors has now shown how conformational changes are transmitted through alpha and beta chains (79). Briefly, in their resting state, integrins are kept in a bent conformation with the head piece in close proximity to the transmembrane part of the alpha and beta chain, and close to the cell membrane. Upon activation, there is a Swiss-blade like opening of the receptor to take a more elongated state (Figure 1). Studies on the integrin α_L_β_2_ identified the β_2_ chain I-like domain, with structure highly resembling the I domain, as critical in forming a contact to the C-terminal helix of the α_L_I, thereby exerting a pull sufficient to open the conformation of the I domain (80). A similar mechanism would be expected for both CR3 and CR4. The critical interplay between the alpha and beta chains in transmitting the conformational signal to regulate ligand binding was demonstrated earlier by studies on CR4.

CR4 is probably the most difficult CD18 integrin to activate. As one part of the challenge to enable ligand binding by CR4, it should be noted that CD18 integrins in most expression system require co-expression of both the alpha and beta chain to be presented on the cell surface or secreted in a well-folded state. The reasons for this requirement are not clear, although it may be speculated that the chains exert a mutual, and critical, chaperone-like activity, which ensures that only correctly paired heterodimers reaches the compartments for CD18 integrin function. In principle, mutations in the human chains could enable activation, but prior to the detailed structural information now available, such a strategy would face the dual problem of making constructs that enabled ligand binding and maintaining sufficient integrity to permit heterodimer formation. Bilsland et al. (81) tested the elegant hypothesis that co-expression of the human α_X_ chain with the chicken β_2_ chain would produce an expressible construct with sufficient alterations in the pairing between the two chains to enable ligand binding activation. In effect, since this construct bound iC3b, while a construct with the native human chain did not, is clear evidence that the alpha-beta chain pairing is important in regulating the activity of the CD18 integrins. This is also of direct consequence to the studies on the ligand binding sCD18 species, discussed in Section Soluble CD18 complexes Evans et al. (44) noted that, in the case of sCD11a/CD18, portions of the alpha chain was probably degraded, but ICAM-1 binding activity was retained. With the insight from CR4 on how contacts between the alpha and beta chains restrain activation (81), it seems likely that proteolytic removal of some alpha (or beta chain) domains would unleash the ligand binding activity of the soluble ectodomains.

**Figure 3:**
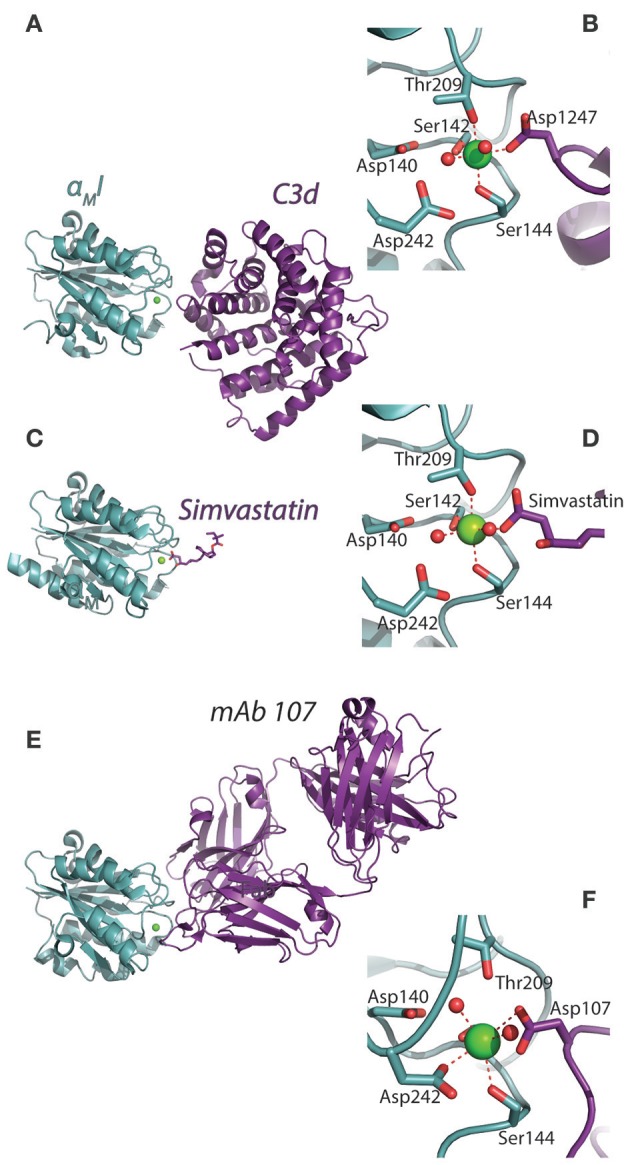
Structural comparison of ligated α_M_I. **(A–F)** By use of XRC, the α_M_I has been characterized in complex with **(A,B)** C3d [4M76; (14)], **(C,D)** simvastatin [4XW2; (83)], and **(E,F)** the ligand-mimicking antibody mAb 107 [3QA3; (84)]. For each complex **(A,C,D)** the corresponding MIDAS arrangement is indicated with the external ligand for the metal ion coordination sphere **(B,D,F)**.

**Figure 4:**
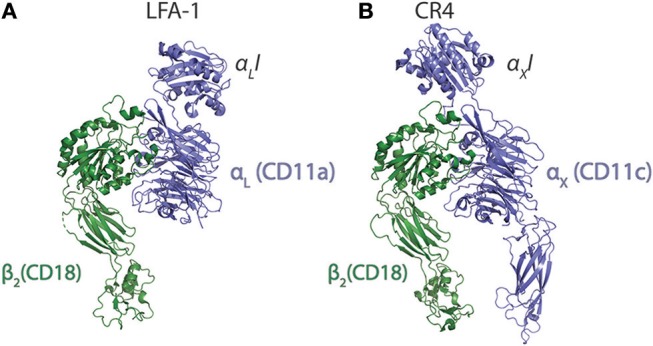
Comparison of the ecto domains of LFA-1 (integrin α_L_β_2_) and CR4 (α_X_β_2_). **(A,B)** The LFA-1 [5E6S; (85)] **(A)** and CR4 [5ES4; (85)] **(B)** are shown with the alpha chain in blue and the beta-chain in green.

CR4 was the first ectodomain of an I-domain carrying integrin to be studied with XRC by Xie et al. (55). In addition to adding further insight to the nature of the conformational lability of the ecto domain, it was clearly demonstrated that the α_X_I is loosely attached to the remainder of the ectodomain body through long loop regions. Xie et al. explained this finding as logically offering some structural freedom in the ability to form contacts with ligands. Indeed, at least on speculative grounds, one would think that such freedom would be usable to solicit further interactions with the beta-propeller domain as experimentally found for the CR3:iC3b interaction. Curiously, however, at least in the case of CR4, there seems not to be such interactions (49). Another possibility for the need of I domain flexibility, if not often addressed in CD18 integrin ligand binding studies, concerns the involvement of the divalent metal ion of the MIDAS in the contact. In the case of C-type lectins, which binds carbohydrate hydroxyl groups through a chelated Ca^2+^ ion, in many respects chemically similar to Mg^2+^, an important paper showed by NMR that the permitted stereochemistry of this interaction constrains the position of the carbohydrate (82). In I domains, the stereochemistry of ligands in the primary coordination sphere of the Mg^2+^ is likely to restrict the movements to pivoting around the chelated anion, similar to what have more recently been observed for the α_M_I:simvastatin complex (see Section Mechanistic basis for αM and αX I domain recognition of structurally diverse ligands). Accordingly, when structures of the ligand or ligand mimetics are compared, the fixed stereochemistry of Mg^2+^ coordination sphere is striking (Figures 3A–D). When the CR4 ectodomain is compared with the ectodomain of LFA-1, it seems that the loop regions connecting either of the alpha chain I domains are longer in CR4 (Figures 4A,B). With the MIDAS requirements for a certain orientation of the Mg^2+^ coordination sphere ligands, the flexible attachment of the α_X_I probably serve to enable successful chelation of acidic groups in ligands even with considerable variation in the structural environment of these groups. The study by Sen & Springer (85) concluded that, at least in the case of LFA-1 and CR4, the I domain flexibility is only structurally limited by the contact with the headpiece platform and that both integrins probably permit large movements of their I domains. However, the LFA-1 carries four sites for attachment of large, N-linked glycosylations in the vicinity of the I domain, while the CR4 has none such. These differences in features could explain how CR4 may bind multiple ligands, even with multiple sites within the same molecule (49, 86–88), while LFA-1 is far more restricted in its interactions.

### Structural insights on CR3 and CR4 ligand binding

Considering the long list of ligands for CR3 and CR4, the number of structural studies on ligand interactions is disappointingly limited. There is no doubt that one fascinating part of CR3 and CR4 biology is how they accommodate binding to such a large inventory of chemically highly diverse ligands. Fortunately, recent progress in structural studies on especially CR3 offers valuable data.

As mentioned above, indirect evidence of the MIDAS function was produced from XRC on the α_M_I. On one hand, these structures indicated an open conformation, which enables the contact with a glutamate side chain from a neighboring domain in the crystal lattice, while the closed conformation would not support such an interaction. On the other hand, in this homotypic interaction, the glutamate was the only contact between the domains, which seemed to exclude this interaction as reflecting a proper protein-protein interaction (65), usually requiring larger surface areas to form stable contacts. This was later found for the α_L_I in complex with ICAM-1 producing a buried surface area of 1,250 Å^2^ (63). Even so, the homotypic interaction is a quite persistent property of α_M_I and CR3. Recent EM studies clearly show that the homotypic interactions also can be found with the CR3 headpiece, in this case forming an abundance of dimers (49). Due to limitations in the structural resolution, this interaction is not clarified at the atomic level.

Two interesting reports detailed the inhibitory potential of the antibody mAb 107, to the α_M_I. Surprisingly, in the authors' terms, the antibody acts as a ligand mimetic (84, 89). mAb 107 stabilized the α_M_I in the closed conformation, even when using α_M_I constructs mutated to favor the open conformation (Figures 3E,F). This stabilization occurred with a Ca^2+^, rather than Mg^2+^, in the MIDAS. Further separating this structure from others complexed integrin I domain structures was the finding of bidentate involvement of aspartate side chains as part of the Ca^2+^ coordination sphere. This work highlights the surprisingly multifaceted nature of the MIDAS in regulating CR3 ligand binding, especially because the structure could have natural, but so far elusive, ligand correlates. From earlier metal ion affinity measurements directly on the α_M_I, it is clear that, in the isolated domain, Mg^2+^ is strongly favored over Ca^2+^, although none of the affinities would permit the MIDAS to be saturated with metal ions at the physiological concentrations (66, 90). This was also found for the α_L_I, where hypo or hyper saturation with Mg^2+^ compared to physiological levels, strongly changed the interaction with ICAM-1 (91). Taken together, this work suggests that the CR3 MIDAS metal ion binding is part of both the conformational dynamics and potentially contributing some regulation of the ligand binding.

**Figure 5:**
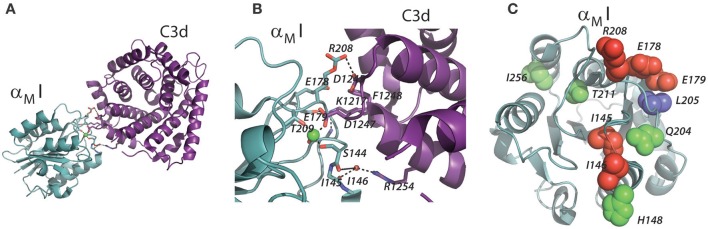
Contacts between the α_M_I and C3d. **(A,B)** The structure of α_M_I in complex with C3d is shown as determined by X-ray crystallography [4M76; (14)]. The α_M_I domain is indicated in turquoise and the C3d fragment indicated in purple. Residues involved in the interaction are represented as sticks, and the polar interactions are indicated by dotted lines. **(C)** The structure of α_M_I with residues involved in the C3d or iC3b interaction shown as spheres. Residues implicated from mutagenesis studies in α_M_I and binding to iC3b are indicated in green (92), residues implicated by XRC (14) are indicated in red, and residues implicated both methods are indicated in blue.

The first structure revealing details of CR3 complement binding was made by Bajic et al. (14), who characterized the complex between α_M_I and C3d (Figures 3A, 5A,B). C3d essentially constitutes the minimal binding site for the domain. The complex interface was formed by an aspartate side chain chelating the MIDAS, occupied by a Ni^2+^ ion available in the mother liquid generating the crystal. As judged from ligand binding measurements by surface plasmon resonance (SPR), this binding site is hidden in the C3b structure, but exposed in iC3b. This is fully consistent with necessity of FI cleavage of C3b to produce the CR3 ligand iC3b (9). With the CR3 binding site located in C3d, the considerable conformational change induced in C3b's conversion into iC3b involving a partial detachment of C3d now offer a structural rationale for the classic characterization of the CR3 recognition of C3 fragments (14, 93). The affinity (*K*_D_) for C3d is in the sub micromolar-range on a par with the α_L_I:ICAM-1 complex, further corroborated by the size of the interface area at 491 Å^2^ (14), also close to the value for α_L_I:ICAM-1 and α_2_I in complex with synthetic collagen-like peptide [Ac-(GPO)_2_GFOGER(GPO)_3_-NH_2_], both at 609 Å^2^ (63, 94).

**Figure 6:**
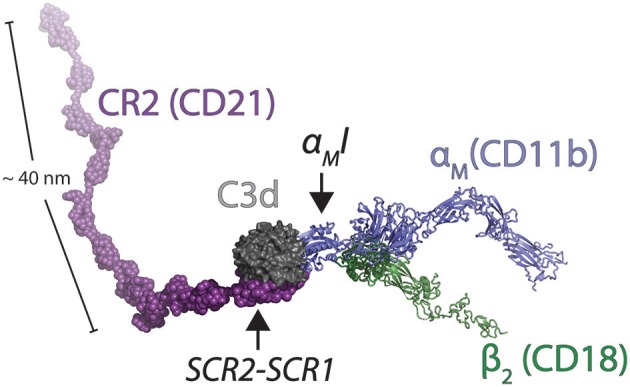
Model of the ternary complex between CR2, C3d, and CR3. The solution structure of CR2 (CD21) was determined by SAXS [2GSX; (95)]. These coordinates were aligned on the CR2:C3d complex [3OED; (96)]. The C3d fragment in the C3d:α_M_I complex [4M76; (14)] was also aligned with C3d in the CR2:C3d complex. To create a model of an extended β_2_ integrin in complex with C3d, the open-conformation α_X_I [5ES4; (85)] was aligned with α_M_I in the C3d:α_M_I complex. The extended conformation of the β_2_ chain was modeled from a structure of the extended β_3_ chain [6BXB; (97)] and the αX chain outside the I domain was modeled from the open structure of integrin αIIbβ3 [2VDN; (98)].

A quite important finding by Bajic et al. (14) was the possibility of CR2 and CR3 to bind the same C3d molecule. CR2 is mainly expressed in B lymphocytes, but is also found in follicular dendritic cells, at least in mice (1). This opens for a quite interesting handling of C3d-opsonized antigens in the lymph nodes. Here, of course, several subsets of CR3-expressing leukocytes reside, including the subcapsular sinus macrophages. As indicated by the name, these cells are in contact with the draining lymph and bordering the leukocyte-dense area of the lymph node, which enables the delivery of antigens to especially B cells. The ability of B of cells to bind the CR3-presented complement-opsonized antigen through CR2, essentially a “hand-over” of antigen (Figure 6), readily extend an important aspect of how the complement system is a part in the formation of antigen stimulation of B cells, and hence antibody formation. With the involvement of CR3 on the cell surface of macrophages, the process becomes essentially an “antigen presentation” to B cells (99). The molecular structures involved are, of course, different from the way antigens are usually presented to T lymphocytes through MHC molecules. On the other hand, it was previously thought that B cell antigen recognition involved mainly events on the B cell surface alone, with complement adding to support the binding through co-binding to CR2 while the B cell receptor engaged an epitope in the opsonized antigen (100). Not excluding the likelihood of these events as well, the CR2:C3d:CR3 complex enables the presentation of antigen in a close contact between the antigen-presenting cell and the lymphocyte. It seems that the large dimensions of particularly CR2 are such that even a quaternary complex with the B cell receptor may be permitted through the C3d-opsonized antigen. It is a classic demonstration that the essentially two-dimensional confinement of juxtaposing receptors in the cell membranes of T cells and APCs greatly enhances the resulting affinity of the receptors for each other (101), compared with a situation where the affinity was measured in (free) solution (102), in effect a three-dimensional compartment. Considering that both the CR2 and CR3 bind C3d with affinities in the micromolar and submicromolar range respectively (103), the principle of 2D affinity is undoubtedly significant in producing results in the cellular context.

### How are CR3 and CR4 capable of binding multiple ligands?

Many reports, with a wide distribution in both time and methodologies, have now shown that CR3 and CR4 bind a vast inventory of ligands (40). Quite a few of these ligands are natural occurring substances, including many proteins, nucleic acids and negatively charged glycosaminoglycans (GAG). In addition, multiple engineered molecules are also on the list, including several peptides and small molecules.

The question on how CR3 and CR4 accommodate such binding has fundamental roots in our understanding on the mechanistic workings of the immune system in at least two ways. First, the concept of immune recognition of the body's foes require a level of specificity in the recognition to avoid undue inflammation in non-infected or otherwise normal tissue. Although the many homeostatic roles of the immune system is now well-established, and hence the need for receptors which can interact with several “self” or altered “self molecules,” receptors on leukocytes should logically be restricted in their ligand binding to avoid autoinflammatory responses. The relevance of such restriction was recently emphasized by the contribution of CR3 to pathological inflammation (104). Nevertheless, at the outset, CR3 and CR4 seems to challenge this concept. Second, it was one of the great scientific accomplishments of twentieth century to explain how “an apparently infinite range of antibody-combining specificity associated with what appeared to be a nearly homogeneous group of proteins” (105) leading to the discovery of the complex somatic genetic rearrangements and mutations encoding these molecules. This is, however, not an option to rationalize how the CR3 and CR4 I domains manage their binding of many ligands, since the I domains are subject to neither genetic nor post-translational modifications. In effect, there is an unresolved matter concerning a type of protein-protein interaction permitting binding of a broad range of ligands.

Below, these questions are further addressed with support from the past two decades of research on the CR3 and CR4 ligand binding.

#### The need for multi-ligand receptors in the immune system

In understanding the functions of CR3 and CR4, it is probably fair to state that there has been a tendency toward placing CR3 and CR4 in the biological context of their ligands one-by-one. Ligands of the coagulation cascade provide an example. Both CR3 (106, 107) and CR4 (108) bind fibrinogen, and CR3 interacts well with its coagulated form, fibrin. Characteristically, there is large number of reports detailing this interaction, identifying the responsible residues in both ligands and receptors (109). In mice, a binding site for CR3 identified in fibrinogen is necessary for the role of this molecule in limiting staphylococcal infections *in vivo* (110). Both CR3 and CR4 were reported to also bind heparin (111, 112), which act to limit coagulation. Add to this list kininogen and plasminogen as CR3 ligands (40), and the receptor would very reasonably seem a part of the wider functions of the coagulation system.

The role in the complement system follows a similar path. There can be little doubt that the C3d fragment is one of the strongest ligands for CR3, hence the crystallization of this ligand—receptor complex (14). The binding site covers a relatively small interface area, which, as mentioned above, is not unusual among integrins and certainly still on par with many other interactions considered specific (113). From investigations of this ligand, CR3 appears a complement receptor in its own right and in ways unrelated to its function in binding coagulation factors.

With these examples in mind, and with a list of many other, less characterized ligands (40), our efforts to understand the multiple ligand interactions by CR3 and CR4 face a significant conundrum from a point of view of structural biology. CR3, and maybe CR4, are clearly able to form classic receptor-ligand interactions, which involves a number of critical side chains in both the receptor and ligand. Nonetheless, among the multitude of ligands reported for each receptor, there is no evidence of any particular shared structural element, at least to a level typical for integrins. Paraphrasing the famous lock-and-key analogy by Emil Fischer (1852–1919) originally addressing the specificity of enzymes but used in many other context of protein interactions since including immunology (114), CR3 and CR4 appear to be “locks” with very definite and distinct structural characteristics, but nevertheless permitting the fit of almost any “key,” in spite of these keys not sharing any obvious similarities themselves.

To understand the immunological relevance of receptors with such properties, it is worthwhile mentioning that one group of receptors seems to share characteristics with CR3 and CR4 with regard to ligand binding, namely the so-called scavenger receptors. Indeed, CR3 has for several years been on the list of scavenger receptors (115). Scavenger receptors, such as CD36, enable cellular removal of decayed macromolecules in extracellular space (116). This decay can be mediated by sources such as oxidation of low density lipoproteins. Both CD36 and the receptor for advanced glycation end products (RAGE) bind many different biomacromolecular coining the designation of multiligand receptors (117). Adding CR3 and CR4 to this group is easily justified, especially as evidence suggest CR3 and RAGE to act in consort with regard to cellular signaling in leukocytes of the innate immune system. A recent paper identified CR3 as reacting with proteins modified by oxidations products of polyunsaturated fatty acids (118). Such modifications as well as several other processes, including proteolysis, impacts protein structure, sometime causing denaturation. This has a special interest in the case of CR3 and CR4, which bind denatured protein well (76, 88, 119). The concept of CR3 and CR4 being scavenger receptors is quite attractive and avoids any too tight association with distinct physiologic processes from simple binding of the associated proteins. The special role of complement, at least in the case of CR3, also fits this proposal well. Complement deposition on apoptotic cells, immunoaggregates and many other plasma-exposed molecular species is a known and important mechanism of cellular clearance (120). Failure of such clearance, for instance through complement component deficiency or defects in CR3, are associated with autoimmune responses such as systemic lupus erythematosus (120, 121). An increasing literature now shows that CR3 outside-in signaling, i.e., the cellular signaling following ligation, serves to down-regulate inflammation by several leukocyte subsets (23). For a receptor on leukocytes involved in clearance of decayed or “altered self” molecular species, both the broad ability to react with many ligands as well as anti-inflammatory regulation are prerequisites for successful—and harmless—completion of this process.

With the many shared ligands, including denatured proteins, it would be simple to claim that CR4 also serve as a scavenger receptor like CR3. There is, however, no evidence that ligand binding of CR4 is anti-inflammatory. Unlike CR3, CR4 is capable of binding highly proteolyzed fibrinogen, increasing the adhesion by neutrophil granulocytes (88). This result was obtained with the proteases plasmin and subtilisin, which mainly share the ability to profoundly degrade many protein substrates. This capability of CR4 was suggested to enable a “danger signal” from proteolytically damaged tissues, for instance as inflicted by certain microbial infections. In such a scenario, there is a coupling between the use, or perhaps more precisely overuse, of a scavenger receptor function and the triggering of a pro-inflammatory response. Other evidence seems to suggest that proteolysis of other ligands may convert these into better ligands for CR4. The role of proteolysis in converting non-ligands into ligands is reminiscent of both the complement and coagulation systems, although these cases usually are being understood as far more regulated. Again, as in the case of CR3 as a scavenger receptor, recognition of, on one hand, highly proteolyzed species and, on another, species probably of multiple origins, would seem to involve a principle quite different from a more standard binding interface in protein complexes.

#### Mechanistic basis for α_M_ and α_X_ I domain recognition of structurally diverse ligands

Surprisingly little effort has been spend on explaining how the CR3 and CR4 recognize structurally diverse ligands, at least compared to the number of reports simply focusing on identifying one or another ligand. As mentioned above, their list of ligands spans not only proteins but also other classes of biomacromolecules, including nucleic acid, GAGs, and lipopolysaccharide (LPS).

One model, which here will be referred to as the “mosaic model” by Ustinov and Plow, embodies the claim from recombinant engineering that the same loop structures on the MIDAS face of the α_M_I apparently are used in recognizing many ligands (122, 123). The logical strength of this model is its classic approach to what is required for formation of a protein-ligand interaction site by clearly providing for a sufficiently large surface area to produce a reasonably strong interaction. As the model predates the α_M_I:C3d structure (Figures 5A,B), the data involved were based on mutagenesis in the MIDAS face of the α_M_I domain. Many, if not all, of the supporting data were generated by mutating selected α_M_I residues into their equivalents in α_L_I, which cannot bind iC3b (122). The lack of binding introduced by these mutations in vicinity of the MIDAS was interpreted as direct engagement of the affected residues in ligand contacts. However, from the mutational investigations on the interaction with iC3b, only one residue was identified, which was also corroborated by the structure of the α_M_I:C3d complex (Figure 5C). This residue was furthermore only involved in a backbone interaction with C3d. As judged from the recent studies by EM, it is unlikely that the α_M_I forms contacts with iC3b outside the C3d fragment (49). The mutational approach probably failed to distinguish direct contacts from indirect loss-of-function through structural alterations of the α_M_I. This prompts a concern over the experimental evidence for the “mosaic model.”

Another model, here named the “anion chelation model,” also makes a starting point with the α_L_I, which is different from both the α_M_ and α_X_ I domains, in so far as the α_L_I has been reported to only bind the structurally highly conserved ICAMs (40). Any model explaining why the α_M_ and α_X_ I domains bind many ligands should, in consequence, also embody the α_L_I in explaining why this, otherwise highly similar domain, will not. A central inspiration is here the above mentioned α_M_I structure with a crystal lattice contact producing a glutamate side chain coordinating the MIDAS of an open-conformation I domain (64) (Figure 2B). Nothing similar was reported for the α_L_I in spite of several available crystal structures (62, 63). In a simple inhibition experiment using surface plasmon resonance, Vorup-Jensen et al. showed that free glutamate acts as an antagonist of fibrinogen binding by α_M_I and α_X_I (88). Calculations on the solution affinity of these domains for free glutamate came to a *K*_D_ of ~2 × 10^−4^ M. Similar experiments with the α_L_I and ICAM-1, estimated the affinity of the α_L_I for free glutamate to be a 100-fold lower with a *K*_D_ of ~2.5 × 10^−3^ M. Similar findings for the α_X_I could be made with compounds such as acetate. This identifies anionic compounds, most likely in the form of carboxylates, being unusually strong ligands for the α_M_I and α_X_I, but not the α_L_I. At the same time, anionic moieties are present in most, if not all, of the reported ligands for α_M_I and α_X_I, pointing to a shared, if yet minimal, structural motif in the ligands of α_M_I and α_X_I.

At this point, support for the anionic chelation model can be found from several sources of investigations.

First, not only will proteins and other macromolecules often carry anionic moieties; these will also be present in different sites within the same molecule, giving rise to multiple binding sites. This was easy to demonstrate with the highly quantitative SPR experiments, where such multiple binding have been directly observable in several experiments from calculations on the moles of immobilized ligand and moles of bound analyte, i.e., α_M_I or α_X_I (86–88, 124). As discussed elsewhere (40, 87), this can furthermore be made as a model-free calculation, which avoids the usual reservations regarding extrapolated values. Any influence from immobilization of the ligand on the stoichiometry by destroying binding sites hardly applies in this context, where the apparent stoichiometry exceeds 1:1. In the case of CR4, the concept of multiple binding sites within a single molecule was recently confirmed by EM, which showed a class of interaction with two recombinant CR4 ectodomains bound to the same iC3b (49).

**Figure 7:**
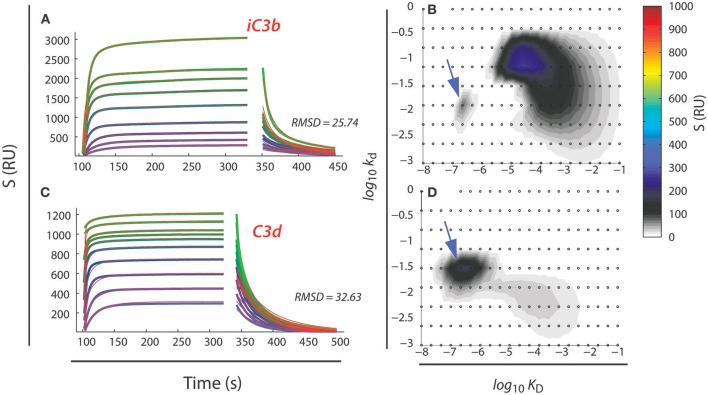
Analysis of SPR data for the α_M_I binding to iC3b **(A,B)** and C3d **(C,D)**. Concentrations of 250 nM to 100 μm of α_M_I (in ascending order of sensorgrams) was injected over SPR surfaces coated with either iC3b **(A)** or C3d **(C)**. Data were analyzed by the EVILFIT algorithm (125, 126), which returns the minimal ensemble of 1:1 binding reactions to account for the experimental data **(B,D)**. Each 1:1 reaction is typified by it dissociation equilibrium constant (*K*_D_ in M) and its dissociation rate (*k*_d_ in s^−1^) with the z-axis (colors indicating resonance units, RU) indicating the abundance of the reactions. In panels **(A,C)**, the experimental data are indicated with a colored lines while the model is indicated with black lines. Deviations between the experimental data and the model were calculated as the root-mean-square-deviation (RMSD). Data from Bajic et al. (14).

A second aspect also derives from the multiplicity of binding sites within a single protein species. While the binding sites share the carboxylated side chains as the central part, nearby side chains or other structural features may still affect binding of the I domain. In effect, this means that the binding kinetics to the sites may differ, producing a heterogeneous interaction between the α_M_I and α_X_I and their ligands. This phenomenon is clearly observable in SPR or similar experiments, where the sensorgrams reflect a composite of binding reactions, unlikely to be accounted for by single exponentials as it would be expected for simple 1:1 reactions. A robust solution to analyzing such experimental data has been provided by Schuck et al. with an algorithm enabling determination of the minimal ensemble of 1:1 reactions required to explain the experimental data set (125, 126). This algorithm has now been applied to analysis of multiple ligands (14, 83, 86–88, 127, 128). Typically, the experimental design used the ligand coupled onto surfaces with the I domains applied in the flow stream. However, in a recent experiment studying the α_M_I binding to the antimicrobial peptide LL-37, it was possible to show that the ensembles determined in the reverse orientation with immobilized I domain were equivalent to those with I domain in the flow stream (128). An example of the interaction between α_M_I and iC3b and C3d are provided in Figures 7A–D. From the analysis, it is clear that the compactly folded C3d provides an almost homogenous interaction (Figure 7D). Although C3d presents more than one carboxylate on its surface, the compact folding would limit the access to the relevant side chains. As expected, this interaction is discernible in the ensemble of interactions characterizing also iC3b (Figure 7B). However, the much larger iC3b molecules, with several regions less compactly folded than the C3d part (93), provides additional types of interactions, in particular some with a *K*_D_ at 10^−4^ M, i.e., close to the *K*_D_ for the interaction with free glutamate (Figure 7B). Similar findings have been made for fibrinogen (88) and the intrinsically unstructured myelin basic protein (MBP) and the likewise unstructured antimicrobial peptide LL-37 (127, 128).

**Figure 8:**
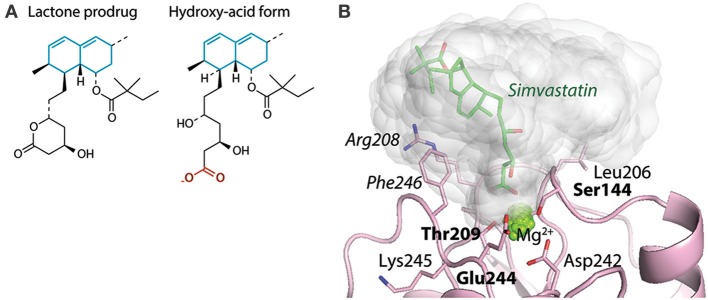
Interactions between the base of simvastatin hydroxy-acid and α_M_I. **(A)** The structures of simvastatin in the lactone prodrug and (base of the) hydroxy acid forms. The unsaturated decalin ring is indicated in cyan, and the carboxylate of the hydro-acid form is shown in red. **(B)** With molecular dynamics simulations, the occupancy of simvastatin is indicated as the covered volume (in gray) together with one simvastatin molecule (shown as sticks) at the position found by XRC [4XW2]. This research was originally published in the *Journal of Biological Chemistry* (83). The American Society for Biochemistry and Molecular Biology.

A third consequence of the anion chelation model suggest that at least carboxylates would be ligands for the α_M_I and α_X_I irrespective of their “mounting.” It was already shown that acetate and propionate inhibited CR4 ligand binding as efficiently as glutamate (88). This was further confirmed by structural studies over the interaction between the cholesterol-lowering drug simvastatin and the α_M_I (83). Interestingly, while the binding between the simvastatin carboxylate (Figure 8A) and MIDAS Mg^2+^ is relatively stable and almost fixed in geometry as discussed above (Section Structure of CR3 and CR4 ectodomains and Figures 3C,D), both the molecular dynamics calculations and the lack of resolution of the simvastatin decalin ring in XRC point to rotation of other parts of this ligand when it is chelated to the MIDAS (83) (Figure 8B). Further calculations showed that this rotation acts to solicit interaction with side chains in vicinity of the MIDAS. The possibility of soliciting interactions in the MIDAS area through such rotation may contribute necessary binding energy, in particular for small molecules or those ligands not forming a large number of interactions.

Fourth and finally, an important question pertain to if interactions with *K*_D_s at ~10^−4^ M play a role in cellular adhesion. Other single-amino acid interactions, such as between plasmin and lysine, take values a 100-fold lower, in the μM range. However, here it is necessary again (see also Section How are CR3 and CR4 capable of binding multiple ligands?) to consider the special situation governing membrane-bound receptors as discussed by Vorup-Jensen (40). A well-established case is the interaction between CD2 and LFA-3. Measured in free solution, similar to the experiments with α_M_I and α_X_I, CD2 and LFA-3 bind each other with a *K*_D_ at ~1.5 × 10^−5^ M (129). When the receptors are confined in the membrane, however, the principle of 2D affinity applies (101). Although the 2D affinity constant (2D *K*_D_) with units in molecules·μm^−2^ is difficult to compare with the solution-based *K*_D_, a point in the studies is, that the weak interaction as measured in solution translated into ~90% binding saturation when the CD2-expressing T lymphocytes adhered to the LFA-3 expressing surfaces (101). By analogy, the apparently weak interaction between α_M_I and α_X_I and some of their ligands, as recorded in solution by SPR, is probably strong enough to support meaningful molecular interaction between surface-confined receptors and ligands. It also needs to be taken into account that the interactions from clustering of the receptors in the membrane (130) gain a polyvalent structure, which may further strengthen cellular adhesion even with weak, monovalent interaction as the basis (40).

Concerning the physiological significance of the CR3 and CR4 binding of carboxylates, a somewhat overlooked aspect also involves the availability of free glutamate for α_M_I and α_X_I binding. In plasma, the free glutamate concentration is ~100 μm, or 50% of the *K*_D_ of α_M_I and α_X_I for this compound (131). From first principles in chemistry, one would expect a 33% saturation of open-conformation I domains. Even more compelling for at least an occasional role of free glutamate in binding these receptors, in experimental models of staphylococcal brain infections, it was shown that glutamate released from damaged neurons increases the cerebrospinal fluid concentration to 500 μm, corresponding to 70% saturation of the I domains of CR3 and CR4 expressed especially on microglial cells (131). How and when free glutamate affect CR3 and CR4 remains unexplored.

#### Ligand binding selectivity of CR3 and CR4

**Figure 9:**
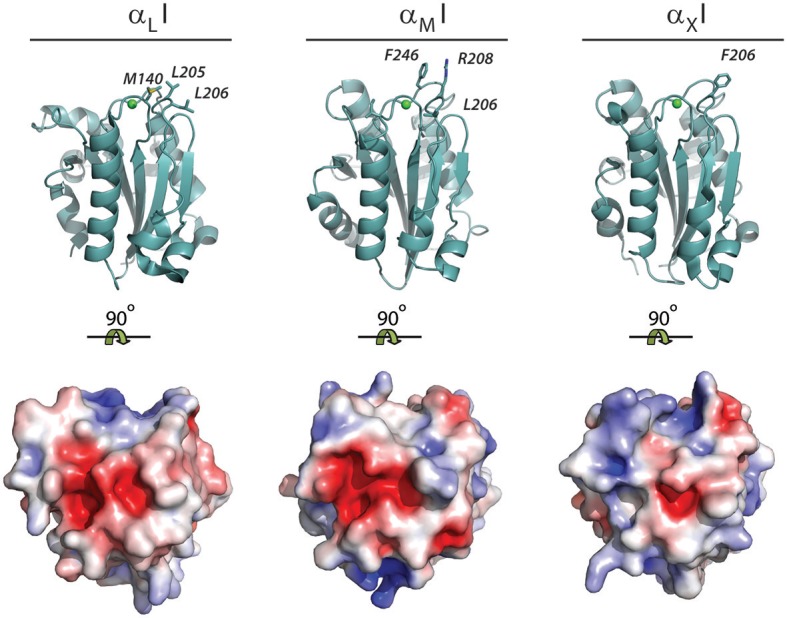
Hydrophobic residues and electrostatic charge in the MIDAS binding interface of the α_L_I, α_M_I, and α_X_I. The structure of the open conformation of the α_L_I [1MQ9; (63)], α_M_I [1IDO; (65)], and α_X_I domain [4NEN; (132)] as determined by XRC. The structures are either represented as cartoons with the hydrophobic residues near the MIDAS binding interphase shown as sticks or shown as the water accessible surface with the electrostatic surface potential represented from−5 kT/e^−^ (red) to 5 kT/e^−^ (blue). The electrostatic surface potential was calculated using Adaptive Poisson-Boltzmann Solver using standard parameters (133). Due to the difficulties in calculating the electrostatic potential of coordinated divalent metal ions (134), structures were modeled without such, which makes the electrostatic charge of the unoccupied MIDAS negative.

From the discussion above, it would be tempting to conclude that the presence of carboxylates is the single most important property characterizing the ligands of α_M_ and α_X_ I domains, and hence CR3 and CR4. Indeed, a standard negative control on integrin involvement in any binding involves testing the binding in a buffer containing EDTA, removing the MIDAS Mg^2+^ ion. However, with the crystallization of the α_X_I, enabling a direct structural comparison with the α_M_I, it became clear that their surfaces in vicinity of the MIDAS are quite different with regard to their presentation of hydrophobic elements and electrostatic charge (Figure 9). Notably, the α_X_I present a ridge of positively charged residues, which is not found in the α_M_I (68). This may well explain some of the ligand binding differences now reported between these domains.

Vorup-Jensen et al. found that the affinity of α_X_I for polyglutamate was higher than for free glutamate (88). Although chemically very different, heparin sulfate also binds the α_X_I strongly (88, 112). Heparin is a random co-polymer of repeating disaccharide residue of d-glucosamine and uronic acids with varying levels of sulfation, making heparin amongst the most negatively charged compound in the human body. It is unclear if sulfate groups may act as ligands for the MIDAS. However, in experiments with purified heparin fragments, there was a clear correlation between the level of sulfation of heparin fragment and their affinity for the α_X_I. By contrast, the α_M_I showed a relatively poor affinity for these species (112). Another highly negatively charged molecule is osteopontin (OPN) (135). The negative charge is contributed both by aspartate and glutamate residues as well as multiple phosphorylations. Surprisingly, the phosphorylation seems to play no role in the interaction with the α_X_I (86, 136). This leaves the high density of negatively charged side chains as the primary source of polyanionicity. In addition to the work with isolated α_X_I, the preference for such ligands was also demonstrated with the intact CR4 in cell membranes (86).

In striking contrast, polyanionic molecules do not bind well α_M_I or the intact CR3. Rather, evidence suggests a much better interaction with cationic species. For instance, MBP, thought to constitute an important autoantigen in MS, binds CR3 (127). Due to its role in forming contact with negatively charged phospholipid membranes, it carries a high positive charge with a resulting pI of 10. The antimicrobial peptide LL-37, a highly positively charged proteolytic split product from human cathelicidin, is also a ligand for CR3 (128, 137, 138) with an affinity comparable to C3d (128). These reports are further supported by a recent analysis suggesting a degenerate protein motif of positively charged residues as binding α_M_I (137).

The difference in ligand binding selectivity seems to add a complementary aspect to CR3's and CR4's function. Both polycationic and polyanionic species are fairly abundant species in the body. Cationic molecules often seem to share a significant role in interacting with cell membranes, which could enable functions of a receptor clearing such species in situations where membrane damage is involved. As one example, we have already suggested that CR3 serves a role in interacting with damaged oligodendrocyte membranes in a manner, which could, however, be exacerbated as part of MS pathology (127). A receptor such as CR4 recognizing polyanioinc species may serve quite different functions. Many microbial organisms carry a high negative surface charge, contributed by cell surface constituents such as peptidoglycan and LPS. As well-known from development of nanoparticles, negative charge add to the colloidal stability (139). In this way, the negative charge on microbial organisms become a pattern in the sense of Janeway's concept of innate immunity, namely a chemical trait that the microorganism cannot survive well without and may serve for recognition by the immune system by germ-line encoded receptors (140). The duality between scavenger and immune receptors noted by Gordon (115) also applies here. Many plasma proteins, including fibrinogen, carries a net negative charge. Making these negative charges more accessible through damage to the protein structure, prompts CR4 recognition. Such damage is a consequence of both normal physiological processes such as coagulation as well as excessive proteolysis induced by microbial organisms, most notably bacteria procuring amino acids from the environment. In this way, a receptor with the ligand binding preferences of CR4 will act as both a scavenger to collect damaged proteins as well as potentially alerting the immune system to microbial threats (88).

## Therapeutic interventions targeting CR3 and CR4

Currently, several immunomodulatory therapies aim to manipulate the function of receptors in the cell surface of leukocytes. They usually induce immunosuppression to reduce symptoms in inflammatory disease or, more recently, supporting immune activation, which enable the elimination of malignancies. A large number of both *in vitro* and *in vivo* experiments suggest that therapeutic targeting of both CR3 and CR4 could potentially produce effects such as lowering of autoimmune inflammation (141) or enhance the effects of anti-cancer vaccination (142). Even so, apart from a clinical trial aiming to improve the outcome in stroke by blocking the function of CR3 (see below), to our knowledge no other attempts were made to target either CR3 or CR4. It is beyond the scope of the present review to discuss the pharmacological and clinical challenges in doing this. Below, a perhaps more surprising point is made, namely that both CR3 and CR4-binding molecules are almost routinely used in current medical treatments. This is, of course, a consequence of the broad range of ligands bound by these receptors discussed in Section The structure, conformational regulation and ligand recognition by CR3 and CR4. In an era where drug repurposing is increasingly seen as a convenient way to improve therapy without the costs of full-scale clinical trials, the short list of drugs made below is meant as a thought-provoking tool box on how to hit some of the arguably most versatile receptors in the immune system.

### CR3 as target in clinical trials and target for multifunctional drugs

With only one, failed, clinical trial attempting to block the function of CR3, our possibilities of knowing the impact of such therapy is very limited. Below, two other examples of clinically used formulation that may hit CR3 functions, namely glatiramer acetate (GA; Copaxone™) and simvastatin is brought up. Detailed insight on their CR3-directed functions *in vivo* is not available, but *in vitro* experiments may still provide functional evidence on their known anti-inflammatory properties involving CR3.

#### Neutrophil inhibitory factor in amelioration of stroke

It is increasingly evident that inflammatory responses play a significant role in adding to the morbidity of stroke. The role of ischemic reperfusion injuries in stroke has long been clear, while the molecular details of the complement system in aggravating such diseases is rather recent (143). Particular diseases of the central nervous system have benefitted from the use of magnetic resonance imaging (MRI) scanners. Recently, a study using ultra-small super paramagnetic iron oxide (USPIO) particles demonstrated that macrophages, or macrophage-like cells, in the stroke lesion are activated, and additional literature point to these CR3-positive cells as being aggravators of the disease (144, 145). Indeed, early studies in a rat model showed that administration of antibodies against CR3 lessened symptoms of experimentally induced strokes (146) and CR3-deficient mice are less susceptible to such injury (147). In humans, clinical trials were made with the compound UK-279,276, a recombinant analog of the hookworm protein neutrophil inhibitory factor (NIF). NIF is a relatively specific inhibitor of CR3 (148) and bind the α_M_I (149). In this way, the trials with UK-279,276 became the first, and to our knowledge the only, study to aim for direct inhibition of CR3. Although interactions with CR3 apparently was discernible in both preclinical models and in humans (150), the trials supported no evidence, unfortunately, of any benefit in stroke therapy (151). A significant reason was liver clearance and the formation of inhibitory antibodies to this non-human protein (150, 151). However, the study suggests that targeting of CR3 is well-tolerated, pointing to other pharmaceutical agents as a way forward.

#### Glatiramer acetate as an antagonist of CR3 function

GA is an effective drug in treatment of relapsing-remitting MS (152). It was among the earliest such treatments, in many ways paving the way for later anti-inflammatory therapies used for this disease. The drug itself is among the most complex formulations on the market. The active ingredient, glatiramer, is a mixture of random copolymers made from bulk synthesis by polymerization of the acetic anhydrides of glutamic acid, lysine, alanine, and tyrosine. After polymerization, chromatography is used to provide a heterogeneous mixture of copolymers with a narrow distribution in *M*_r_ around 8,000, or 50–60 residues. This leads to the astonishing observation that the formulation, in principle, may contain any of 10^30^ different co-polymers, while the pre-filled syringes with 20 mg only delivers 10^17^ such co-polymers (153). Although the theoretical number of co-polymers in the clinical formulations may be curbed by complex aspects of the polymerization process (154), there is little doubt that the patients rarely, if ever, receives the same co-polymer twice. The ratios of amino acid anhydrides were mixed to mimic the properties of MBP, one of the used autoantigens in experimental autoimmune encephalitis (EAE), and maybe an autoantigen in human MS as well (153). In effect, this means that GA also carries an excess positive charge from the high abundance of lysine residues. GA is capable of inhibiting EAE and reduces the frequency of attacks in relapsing-remitting MS with ~30%. The pharmacological mode of action (PMA) remains enigmatic. Strong support for the copolymers acting as activators of a polyclonal Th2 type response has been provided (155). T cell proliferation may proceed even in the absence of professional antigen-presenting cells, suggesting that other, extracellular loading of MHC II molecules is a possibility. Stapulionis et al. considered the potential role of CR3 in contributing to the PMA of GA (127). Both cellular adhesion to MBP and iC3b were inhibited by the addition of GA to the medium in a concentration of 3 μg/ml. This nicely matches the resulting concentration from a distribution of the applied clinical dosage of 20 mg in 6 liters of plasma. In agreement with difference in ligand binding selectivity discussed above, CR4 was not capable of binding GA. Experiments with the α_M_I in SPR with the methodologies mentioned above showed a *K*_D_ of ~10^−4^ M. Finally, circular dichroism spectroscopy suggested a significant portion of unfolded polypeptide sequence in GA. Taken together, the mode of CR3 interaction with these co-polymers is probably very similar to the vast range of peptides reported to bind this receptor, with the binding supported by the unfolded character of at least some of the material. CR3 was already shown in animal models to be a factor in development of EAE (141). More recent evidence suggest that onset of the PMA in MS is fast, within hours of the first injection (156). This points away from the adaptive immune response as responsible for all of the effects in MS. However, GA is a complex drug, as demonstrated by the recent observations that it may directly kill T lymphocytes in process similar to LL-37, which is likewise known to possess immunomodulatory properties (157). The cytotoxicity toward prokaryotes only expands the possible therapeutic influences in MS (158). However, with the significant role of CR3-expressing macrophages and microglial cells in the pathogenesis of MS, the role of GA as a CR3 antagonist should not be overlooked as part of the PMA.

#### Simvastatin as ligand binding kinetic-dependent antagonist of CR3

In pioneering studies by investigators from Novartis, it was demonstrated that lovastatin inhibited the function of LFA-1 (159, 160), thereby limiting T cell proliferation. Interestingly, the mechanism in this molecule involved a stabilization of the α_L_I in the closed conformation by the binding of the statin to the so-called L-site away from the MIDAS. This allosteric antagonism came as a surprise, since lovastatin in its activated form presents a carboxylate, which, from the findings mentioned earlier on α_M_I (159), was expected to chelate in the MIDAS. Jensen et al. (83) investigated the interaction between the open-conformation α_M_I as well as activated CR3 receptors. In both types of assays, simvastatin, a compound highly similar to lovastatin and also an antagonist of α_L_I (159), inhibited the CR3 binding to iC3b, in the cellular experiment with an IC_50_ in the order of 10 μm, similar to IC_50_ for inhibition of LFA-1 to ICAM-1. The simvastatin carboxylate (Figure 5A) was firmly chelated in the α_M_I MIDAS (Figure 5B), clearly advocating that the statin in this case acted as a competitive antagonist. A more detailed analysis was provided in SPR studies. With iC3b as a ligand, the total inhibition was limited, but discernible. Surprisingly, no inhibition was found with ICAM-1 as a ligand for α_M_I, a result also supported by earlier, but unexplained, findings (160). Closer inspection showed that inhibition of iC3b binding quantitatively came from a relatively select elimination of interactions with slow association and dissociation rates, while other types of interactions were left unaffected. Accordingly, the slow-binding-kinetic type of interaction was not found in the binding to ICAM-1, explaining why this ligand was not affected. It is not uncommon to find larger antagonist involved in complex binding schemes as recently demonstrated by the allosteric mechanisms of natalizumab, a MS drug, in antagoniszing the T cell adhesion molecule very-late antigen (VLA)-4 (161). However, that small-molecule drugs also seem to be capable of participating in complex inhibition reactions is more surprising with only speculative explanations provided so far.

### CR4 as a drug target and what it may help

With the more mysterious role of CR4, one should think it is difficult to identify clinically relevant inhibitors or other compounds influencing CR4 functions. Surprisingly, three examples can be extracted from the literature, one involving highly sulfated heparin fragments, another focusing on a food additive, OPN, which has been suggested to stimulate the immune system, and finally a potential relationship between adjuvants and CR4.

#### Heparin as a CR4 ligand binding antagonist

Although both CR3 and CR4 were reported to support adhesion to heparin, a quantitative measurement with side-by-side comparison of the inhibitory potential of heparin fragments, clearly suggested that CR4 is the better receptor for heparin (112). As mentioned above, the affinity was strongly influenced by the level of sulfation, with higher sulfation strengthening the interaction. Likewise, the length of the heparin oligomers was important. Natural heparin has a degree-of-polymerization (dp) of ~42 and was a strong inhibitor of CR4 with IC_50_ at 0.30 μm. Heparin with a dp21 (*M*_r_ ~ 6,000), similar to the low-molecular weight heparin used in the clinic (162), had a IC_50_ of 0.1 μm (0.6 mg/l) (88). This should be compared with the subcutaneously injected dosage of 1 mg/kg body weight (162). The simple calculation does not address the complex issue of distribution volume, but it seems not impossible that clinical injections of heparin may reach a concentration sufficient to impact the function of CR4. This should be compared with the effect of fondaparinux (Arixtra™), a pentameric, artificial heparin-like compound, which is capable of preventing coagulation (163). It showed no quantitative interaction with CR4 (112). This opens an interesting perspective on how to design artificial heparins. Accelerated by the so-called “Heparin crisis” in the early 2000s, where contaminated heparin provoked severe hypersensitivity responses in patients treated with contaminated heparin, a clinical unmet need exists in producing safe, synthetic formulations (164).

#### OPN, immunostimulatory food additive

OPN is a highly phosphorylated protein, which serves roles both in the bone matrix and beyond. It is possible to purify the protein from both human and bovine milk. It is also found in human serum with some association of the concentration with human diseases, possibly suggesting a use of OPN as a biomarker. Its proposed role in immunology mainly stems from association with inflammatory diseases such as arthritis (135). Experimental studies with milk formula enriched in OPN suggested that OPN increases the number of circulating T lymphocytes in formula-fed infants (165). From these and other data, an interleukine-like function seems likely (166). This opens the discussion on what receptors, expressed in leukocytes, are relevant. As already mentioned in Section Ligand binding selectivity of CR3 and CR4, CR4 binds OPN strongly, probably in consequence of the negative charge of this protein contributed by multiple glutamate side chains. CR4 is, however, not the only OPN receptor. It contains an Arg-Gly-Asp (RGD) motif, which has already been demonstrated to mediate interactions with β_3_ integrins, and other integrins have been reported as receptors as well (167). With the high expression of CD11c/CD18 on dendritic cells and the central role of these cells in regulating intestinal cytokine levels and leukocyte proliferation, notably T lymphocytes, it seems a straight forward proposal that the strong CR4 binding of this molecule play a role in these observations.

#### CR4 as a target in vaccination

An emerging literature has pointed to CR4 as an important target in vaccination (168–170). As noted in Section Functions of CR3 and CR4 and the family of CD18 integrins, CR4 (CD11c/CD18) has long been established as a marker for dendritic cells with a particular high expression in murine dendritic cells (15). With its role as a complement receptor, CR4 is probably significant in the phagocytic uptake by these cells. In principle, this would enable the presentation of peptides from the phagocytozed antigen on MHC II molecules to CD4+ T lymphocytes. However, as shown by Castro et al. antigens conjugated to antibodies to CD11c are capable of raising a CD8+ T lymphocyte response (168). The therapeutic advantages of such a response includes T cell targeting to cancer cells or intracellular infections difficult to limit with an antibody response. Interestingly, the mechanism here seems to rely on cross presentation by dendritic cells, that is, presentation of phagocytozed proteins on MHC I molecules. Although promising, CD11c-targeted vaccines has not formally been tested in humans.

From what we now know of the protein binding properties of CR4, it is possible to ask the question if CR4 already is a part of vaccine responses. Jalilian et al. reviewed the use of adjuvants, mainly in influenza vaccination (171). In spite of some adjuvant formulations having been used for almost a 100 years, we know surprisingly little about their therapeutic effects. The particulate nature of these compounds, notably aluminum salts, seems to suggest that protein deposition on such surfaces could play a role in their interaction with antigen presenting cells vis-à-vis the expression of CR4. The deposition of complement and fibrinogen/fibrin would probably occur more efficiently on the particle-embedded antigen than for the free antigen. In addition, the complex processes leading to denaturation of particle surface-adsorbed proteins apparently further aids the interaction with CR3 (172) and possibly CR4 from binding to such material. Finally, it is well-known that receptor-mediated phagocytosis requires a particle size of about 50 nm, which is a hard-to-reach dimension limit by applying soluble antigens. Taken together, this evidence suggests that a valuable direction of optimizing vaccine adjuvants would include a closer examination of CR3 and CR4 in this context (171).

## Conclusion: CR3 and CR4, significant contributors to both innate and adaptive immunity

Are CR3 and CR4 simple double-ups in the leukocyte cell membranes? Three conclusions from the literature answer this question in the negative.

First, although both receptors unquestionably bind the C3 fragment iC3b, it is very clear that the principle of recognition and bindings sites in use are non-overlapping. Structural and functional analyses do not suggest any striking similarity as to what make CR3 and CR4 complement receptors. iC3b is a large, multidomain protein, which binds a plethora of different proteins, and accommodates all the critical features that enable binding by both receptors. Undoubtedly, this may support an altogether stronger affixing of complement-opsonized antigen to any particular myeloid cell surfaces.

Second, a striking property of both CR3 and CR4 is the large number of reported ligands, some distinct for each receptor, others shared. When comparing with LFA-1, which binds essentially only ICAMs, several sources of experimental evidence suggest that CR3 and CR4 indeed share a stronger ability to chelate carboxylate groups. Maybe for this reason, they also bind denatured or natively unfolded species better than their folded counter-parts. On the other hand, we know little about the strength of carboxylate chelation in the integrin family, as to our knowledge this has so far only been investigated in comparison of the α_M_I, α_X_I, and α_L_I. From the long known ability of the beta-chain I-like domain in β_1_ and β_3_ integrins to bind the minimal RGD motif, it is tempting to suggest that CR3 and CR4 are less unusual encounters in the integrin world than LFA-1. In other words, their ligand binding promiscuity is less of a unifying trait than otherwise could be thought.

Third, even if CR3 and CR4 share ligands, it is now possible to rationalize a different ligand binding selectivity. For more than a decade, it has been clear that polyanionic species, including both negatively charged carbohydrates and proteins are particularly strong ligands for CR4. By contrast, both studies over individual ligands as well as more systematic analyses, suggest that CR3 has preference for cationic species in so far as these may also offer a carboxylated moiety for chelation of the MIDAS. In this sense, CR3 and CR4 nicely fits with properties earlier attributed to scavenger receptors, where at least some also accommodate the binding of homogenously charged species, reflecting a decayed state of macromolecules, including charge exposure of denatured states of proteins.

With these properties of ligand recognition, one surprising observation embodies the clinical formulations in use, which may affect the function of CR3 and CR4. From simvastatin, one of the most often used drugs in the world, to heparin, a classic anticoagulant, *in vitro*, evidence suggests an impact of these drugs on the human immune system. Systematic studies over this impact are lacking, however, probably in part because we need a better understanding of the pharmaceutical benefits from drugs targeting CR3 and CR4. With the recent findings of the roles of complement, including both CR3 and CR4, in NK cell biology, as part of the use of therapy with monoclonal antibodies, these topics are likely to become of high significance.

CR3 and CR4 entered immunology almost a decade before the concept of innate and adaptive immunity was coined. Between them, they expand on both classic and more enigmatic ideas of the function of the immune system. With their functional similarity as complement receptors for C3 fragments, as well as their primary expression in myeloid leukocytes, it is justified to place their role in the innate immune system. However, at least in the case of CR3, its ability to bind C3d-opsonized antigens in conjunction with CR2 on B lymphocytes, highlights a well-established theme of complement as the primer of antibody formation. In addition, it is not without interest that this interaction seems to also highlight another recurrent phenomenon in lymphocyte biology, namely the importance of membrane-presentation of antigens to lymphocytes. The role of CR4 is less well understood, yet its abundant expression on dendritic cells places it in the center of modern immunology. From the review made above, a summary of the comparison between CR3 and CR4 points to a consort of receptors, which together embodies both a surprising versatility and complementarity in molecular recognition mechanisms.

## Author contributions

TV-J and RJ wrote the paper and made the figures.

### Conflict of interest statement

The authors declare that the research was conducted in the absence of any commercial or financial relationships that could be construed as a potential conflict of interest.
